# Computer-aided diagnosis of lung nodule classification between benign nodule, primary lung cancer, and metastatic lung cancer at different image size using deep convolutional neural network with transfer learning

**DOI:** 10.1371/journal.pone.0200721

**Published:** 2018-07-27

**Authors:** Mizuho Nishio, Osamu Sugiyama, Masahiro Yakami, Syoko Ueno, Takeshi Kubo, Tomohiro Kuroda, Kaori Togashi

**Affiliations:** 1 Department of Diagnostic Imaging and Nuclear Medicine, Kyoto University Graduate School of Medicine, Kyoto, Japan; 2 Preemptive Medicine and Lifestyle-related Disease Research Center, Kyoto University Hospital, Kyoto, Japan; 3 Department of Social Informatics, Kyoto University Graduate School of Informatics Yoshidahonmachi, Kyoto, Japan; 4 Division of Medical Information Technology and Administrative Planning, Kyoto University Hospital, Kyoto, Japan; Southwest University, CHINA

## Abstract

We developed a computer-aided diagnosis (CADx) method for classification between benign nodule, primary lung cancer, and metastatic lung cancer and evaluated the following: (i) the usefulness of the deep convolutional neural network (DCNN) for CADx of the ternary classification, compared with a conventional method (hand-crafted imaging feature plus machine learning), (ii) the effectiveness of transfer learning, and (iii) the effect of image size as the DCNN input. Among 1240 patients of previously-built database, computed tomography images and clinical information of 1236 patients were included. For the conventional method, CADx was performed by using rotation-invariant uniform-pattern local binary pattern on three orthogonal planes with a support vector machine. For the DCNN method, CADx was evaluated using the VGG-16 convolutional neural network with and without transfer learning, and hyperparameter optimization of the DCNN method was performed by random search. The best averaged validation accuracies of CADx were 55.9%, 68.0%, and 62.4% for the conventional method, the DCNN method with transfer learning, and the DCNN method without transfer learning, respectively. For image size of 56, 112, and 224, the best averaged validation accuracy for the DCNN with transfer learning were 60.7%, 64.7%, and 68.0%, respectively. DCNN was better than the conventional method for CADx, and the accuracy of DCNN improved when using transfer learning. Also, we found that larger image sizes as inputs to DCNN improved the accuracy of lung nodule classification.

## Introduction

Computer-aided diagnosis refers to software that helps clinicians to diagnose disease, and it has the potential to optimize clinicians’ workloads [1,2,3–7]. Computer-aided diagnosis can be divided into software that detects lesions (CADe, computer-aided detection) and software that classifies lesions (CADx, computer-aided diagnosis). However, for CADe or CADx to assist clinicians effectively, they must perform reliable and efficient image recognition. If a method that can better recognize an image is applied to computer-aided diagnosis, its performance can be improved.

Lung cancers are the leading cause of cancer-related death in the United States because they are frequently diagnosed at an advanced stage [8]. Results from the National Lung Screening Trial showed that lung cancer screening by computed tomography (CT) has significantly reduced lung cancer mortality among heavy smokers, but that false positives were problematic, accounting for 96.4% of positive screening results [9]. Another study has indicated that CADe might help radiologists to detect missed lung cancers on CT screening by assisting with image interpretation [7]. Experience with CADe suggests that CADx might help reduce the number of false positives identified by CT during lung cancer screening.

Deep learning is a new technique that is overtaking conventional methods of computer vision, such as hand-crafted imaging feature plus machine learning, and is increasingly being used in CAD [10]. Deep convolutional neural network (DCNN) has attracted the attention of researchers since its introduction in 2012 at the IMAGENET Large Scale Visual Recognition Challenge [11]. The DCNN method has continued to improve, and it has been shown that image recognition by DCNN was identical or superior to that by humans in general object recognition [12].

Many studies have used DCNN to improve the performance of CAD [10,13–20,21]. Several studies have also proposed the use of DCNN-based CAD for lung nodules. For example, Teramoto et al. proposed that use of DCNN in CADe could reduce the false positive rate in positron emission tomography/CT images of lung nodules [21]. The results of Ciompi et al. also show that DCNN was useful for CADx, helping to classify lung nodules into six types [19].

In the current study, we focused on developing CADx by DCNN for lung nodules. Our aim was to evaluate the following: (i) the usefulness of DCNN for CADx compared with conventional methodology (i.e. hand-crafted imaging feature plus machine learning), (ii) the effectiveness of transfer learning, and (iii) the effect of image size as an input to DCNN.

## Methods

This retrospective study was approved by the ethical committee of Kyoto University Hospital, which waived need for informed consent. We used a database which were built for previous research of CADx [4,22]. Because the previous studies focused on CADx without DCNN, the purpose of the current study is different from those of the previous studies.

### CT image database

The database contained the CT images and clinical information of 1240 patients who had at least one lung nodule. The CT images were acquired using a 320-detector-row or a 64-detector-row CT scanner (Aquilion ONE or Aquilion 64; Toshiba Medical Systems, Otawara, Japan). CT scan parameters were as follows: tube current, 109 ± 53.3 mA (range, 25–400 mA); gantry rotation time, 0.500 ± 0.0137 s (range, 0.400–1.00 s); tube potential, 120 ± 1.69 kV (range, 120–135 kV); matrix size, 512 × 512 and slice thickness, 1 or 0.5 mm. Lung nodules diagnosed as benign nodules, primary lung cancers, or metastatic lung cancers were selected, and the CT images, final diagnosis, and nodule positions of these nodules were used for development and evaluation of CADx.

### Image pre-processing

The CT images were loaded, and their voxel sizes converted to 1× 1 × 1 mm. In each case, because the position of the center of the lung nodule was available, the CT images including the lung nodule were cropped with a volume of interest set to 64 × 64 × 64 mm (voxels). The cropped CT images were then input for CADx.

### Conventional CADx

From the cropped CT images, feature extraction was performed by rotation-invariant uniform-pattern local binary pattern on three orthogonal planes (LBP-TOP) [23,24,25], which has been successfully used for CADx of lung nodules [3]. The results of LBP-TOP were fed to support vector machine (SVM) with kernel trick (radial basis function) [26]. LBP-TOP had two hyperparameters (*LBP*_*R*_ and *LBP*_*P*_), and SVM had two hyperparameters (*C* and ***γ***).

### CADx by DCNN with and without transfer learning

To utilize DCNN for 2D images (2D-DCNN), the 3D cropped CT images were converted to 2D images. Three orthogonal planes (axial, coronal, and sagittal) were set on the center of the 3D images, and 2D images (64 × 64) in the three orthogonal planes were extracted. At extraction, the sizes of 2D images were converted to *L* × *L*, where *L* was set to 56, 112, or 224. With this image processing, each lung nodule was represented as the three 2D images (size = *L* × *L*). We referred to a pair of these 2D images and the corresponding final diagnosis as a batch. Before feeding batches to DCNN, the pixel value range of the 2D images was changed from −1000, 1000 to −1, 1 by the transformation *y = x/*1000, where *x* and *y* were the pixel value before and after the transformation, respectively.

**Fig 1:**
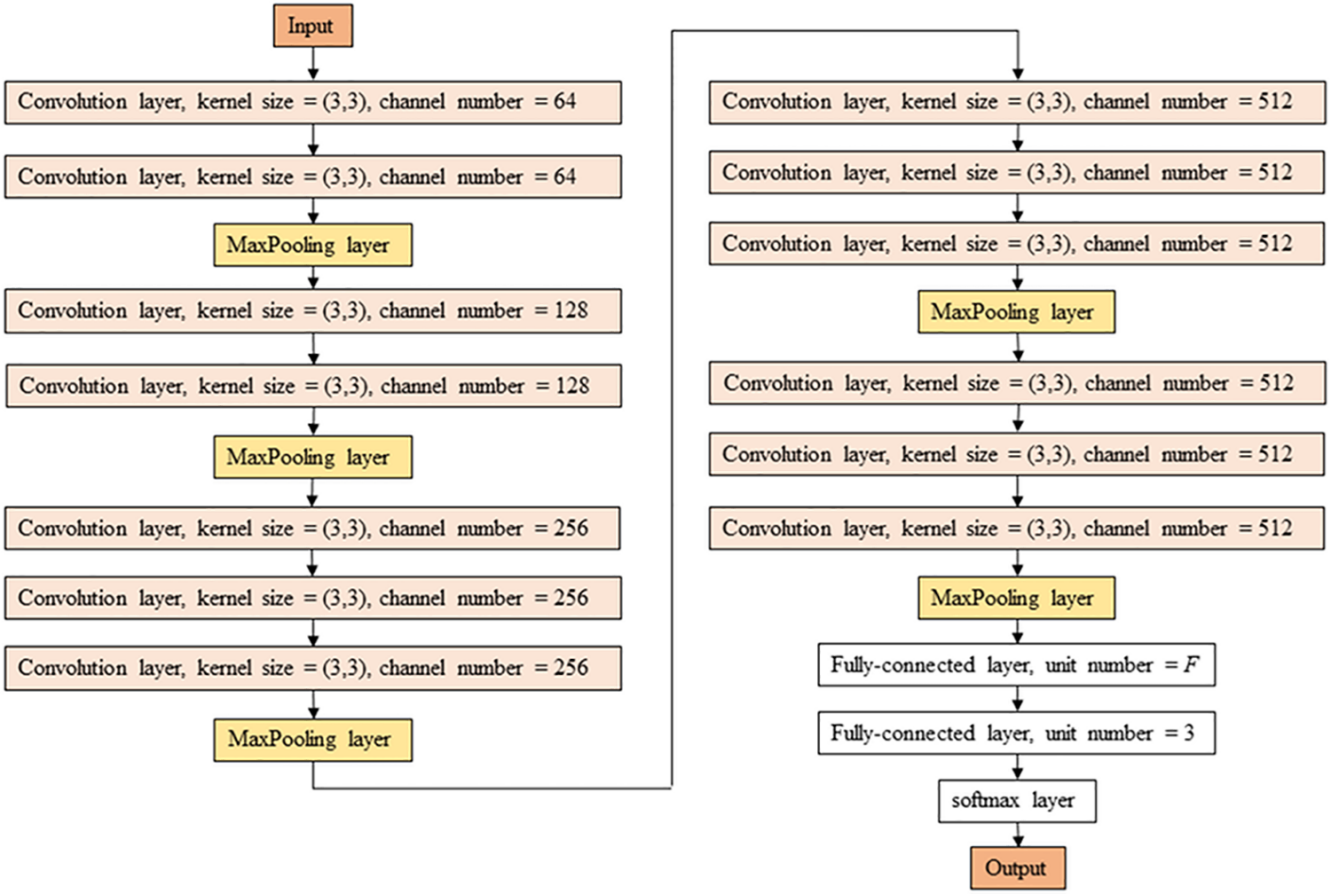
Schematic illustration of the modified VGG-16. Note: Except softmax layer, activation function is not shown.

The architecture of 2D-DCNN in our CADx was derived from VGG-16 convolutional neural network [27], which was modified to perform transfer learning (Fig 1). First, fully-connected (FC) layers of VGG-16 were removed, and a new FC layer was added, whose number of units was denoted by *F*. Next, an FC layer with three units, whose output would be converted to a probability of the three classes, was added as the prefinal DCNN layer. Dropout was applied between the two FC layers, with strength denoted by *D* (0 = no dropout; 1 = full dropout and no connection between the two FC layers). We then used rectified linear units as the activation function of the FC layer with *F* units. To convert the output of the FC layer with three units to a probability of the three classes, a softmax layer was used. For transfer learning, we used VGG-16 parameters pretrained with IMAGENET [11] and finetuned by stochastic gradient descent. The initial learning rate of stochastic gradient descent was represented as *R*. Parameter finetuning was not performed in several VGG-16 layers, and the number of layers without finetuning is represented by *V*. In CADx by DCNN without transfer learning, training was performed without VGG-16 parameters pretrained with IMAGENET. Data augmentation was performed for 2D-DCNN training. Hyperparameters of 2D-DCNN were summarized in Supporting Information.

### Statistical analysis

We used 1113 training cases for learning and 123 validation cases for performance evaluation, which did not overlap. Validation loss and validation accuracy were calculated 10 times with the same CADx hyperparameters [19]; splitting of the training and validation sets was random each time. The averaged values for validation loss and validation accuracy were obtained for each set of hyperparameters and were used to evaluate the performance. For the conventional method, we selected the best LBP-TOP and SVM hyperparameters by grid search [28]. For the DCNN method, we performed random search to optimize the hyperparameters [29]. The detail of random search was described in Supporting Information.

## Results

For benign nodules, primary lung cancers, and metastatic lung cancers, the following number of lung nodules were selected from the database for development and evaluation of CADx: benign nodules, n = 412; primary lung cancers, n = 571; and metastatic lung cancers, n = 253. Four lung nodules were excluded because they did not fit one of these three types (for example, carcinoid). All diagnoses of primary lung cancer were confirmed pathologically. Benign nodules were primarily confirmed by stability or shrinkage on repeat CT scans over a 2-year follow-up period, but 57 were also diagnosed pathologically. Most of the metastatic lung cancers were diagnosed radiologically and clinically, and the diagnosis of 90 metastatic lung cancers was confirmed pathologically. As shown in Table 1, mean and standard deviation of size of these lung nodules were 20.52 ± 10.22 mm.

**Table 1:** Summary of patient demographics.

Variables	All		Benign nodule		Primary lung cancer		Metastatic lung cancer	
	Mean	SD	Mean	SD	Mean	SD	Mean	SD
N	1236		412		571		253	
Age (y)	65.76	12.65	64.81	13.80	68.41	9.70	61.35	14.97
Sex (number of men)	709		237		331		141	
Smoking history (Brinkman Index)	605.1	774.2	543.8	747.7	756.4	841.1	354.0	543.3
Smoking status								
Current smoker	266		70		151		45	
Ex-smoker	456		161		219		76	
Never smoker	514		181		201		132	
Previous history of malignant tumor	545		148		144		253	
Nodule size (mm)	20.52	10.22	18.28	8.54	24.81	10.89	14.48	6.16
Contrast-enhanced CT	531		113		287		131	

Because Brinkman index was not clearly described in 20 patients, Mean and SD of brinkman index were calculated without these 20 patients. Abbreviation: SD, standard deviation; CT, computed tomography.

The current study included 709 men and 527 women, and the patient demographics of these 1236 patients are shown in Table 1. Mean and standard deviation of patient age and smoking history (Brinkman Index) was 65.76 ± 12.65 and 605.1 ± 774.2, respectively. Their smoking status was as follows: current smoker, n = 266; ex-smoker, n = 456; and never smoker, n = 514. Previous history of malignant tumor was confirmed in 545 patients. Contrast-enhanced CT was performed in 531 patients.

**Fig 2:**
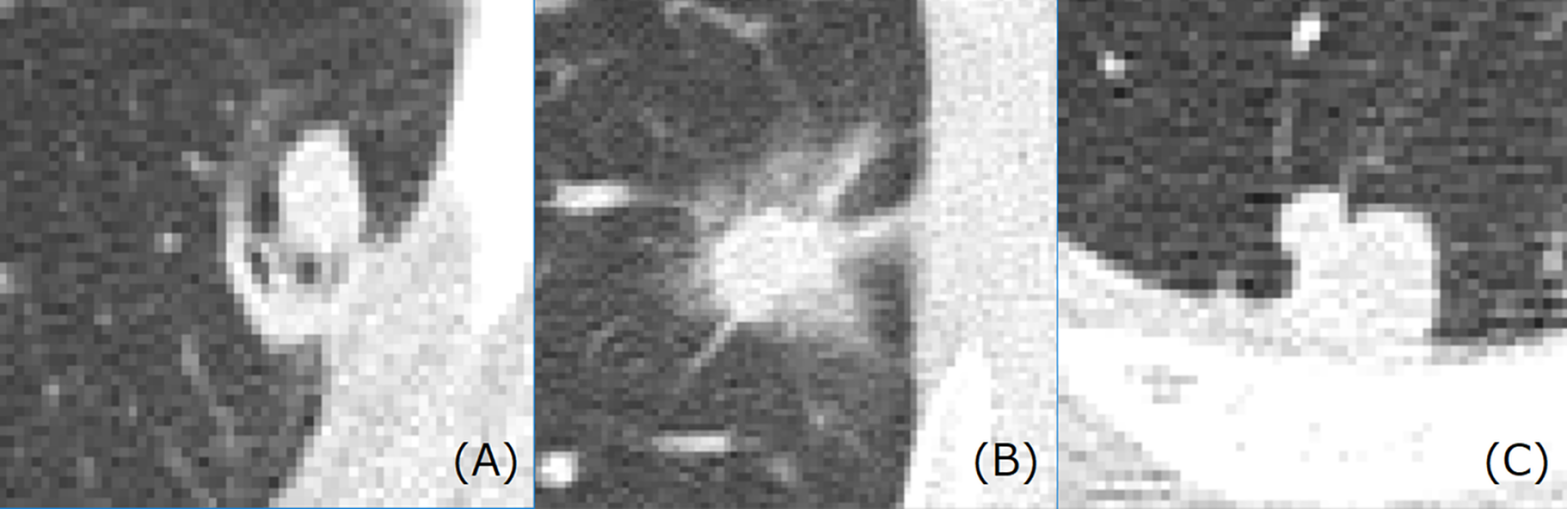
Representative CT images of lung nodules. (A) benign nodule, (B) primary lung cancer and (C) metastatic lung cancer.

**Fig 3:**
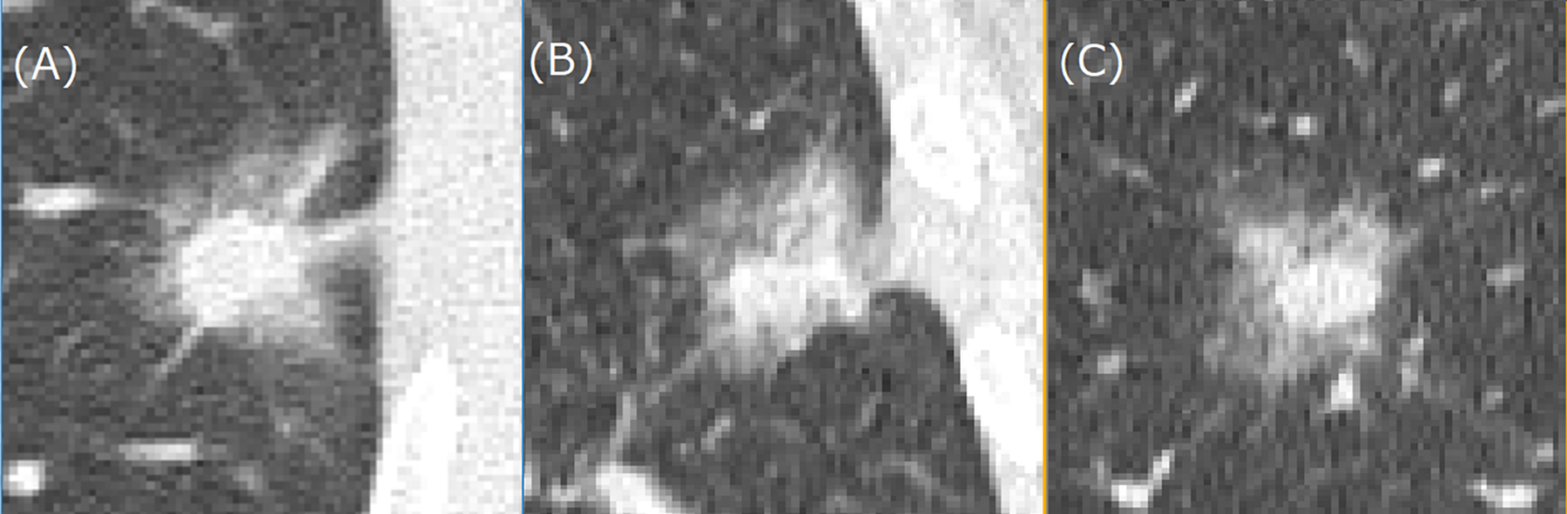
Three CT images obtained from three orthogonal planes used for input to 2D-DCNN. Fig 2(B) is identical to Fig 3(A). (A) axial image, (B) coronal image and (C) sagittal image. Abbreviations: DCNN, deep convolutional neural network.

Fig 2 shows representative CT images of a benign nodule, a primary lung cancer, and a metastatic lung cancer. Fig 3 shows three representative CT images of a lung nodule obtained from the three orthogonal planes and used as the input to 2D-DCNN.

The best averaged validation accuracy for the conventional method was 55.9%, and the following optimal hyperparameters were used: *LBP*_*R*_ = 4, *LBP*_*P*_ = 40, *C =* 1024, and *γ* = 4. Table 2 shows validation loss, validation accuracy, and the optimal hyperparameters for *L* values of 56, 112, and 224 for CADx by DCNN with transfer learning. The best averaged validation loss and validation accuracy for DCNN with transfer learning were, respectively, as follows: 0.822 and 60.7% when *L* = 56; 0.783 and 64.7% when *L* = 112; and 0.774 and 68.0% when *L* = 224. Table 2 also shows validation loss, validation accuracy, and the optimal hyperparameters for *L* values of 56, 112, and 224 for DCNN without transfer learning. The best averaged validation loss and validation accuracy for DCNN without transfer learning were, respectively, as follows: 0.843 and 60.2% when *L* = 56; 0.824 and 62.4% when *L* = 112; and 0.860 and 58.9% when *L* = 224. The raw results for optimal CADx with DCNN are shown in Supporting Information, as are the averaged validation loss and validation accuracy data in all trials of random search.

**Table 2:** Optimal hyperparameters and classification results for CADx by DCNN with and without transfer learning.

Type	*L*	*E*	*R*	*V*	*F*	*D*	Validation Accuracy (%)	Validation Loss
DCNN with TF								
	56	20	0.00002	4	384	0.6	60.7	0.822
	112	20	0.00002	11	384	0.4	64.7	0.783
	224	20	0.00002	11	384	0.4	68.0	0.774
DCNN without TF								
	56	30	0.00007	0	384	0.6	60.2	0.843
	112	25	0.0001	0	384	0.4	62.4	0.824
	224	15	0.0001	0	384	0.4	58.9	0.860

validation loss and validation accuracy were calculated 10 times with the same CADx hyperparameters, and their averaged values were shown. Abbreviations: CADx, computer-aided diagnosis; DCNN, deep convolutional neural network; TF, transfer learning.

**Fig 4:**
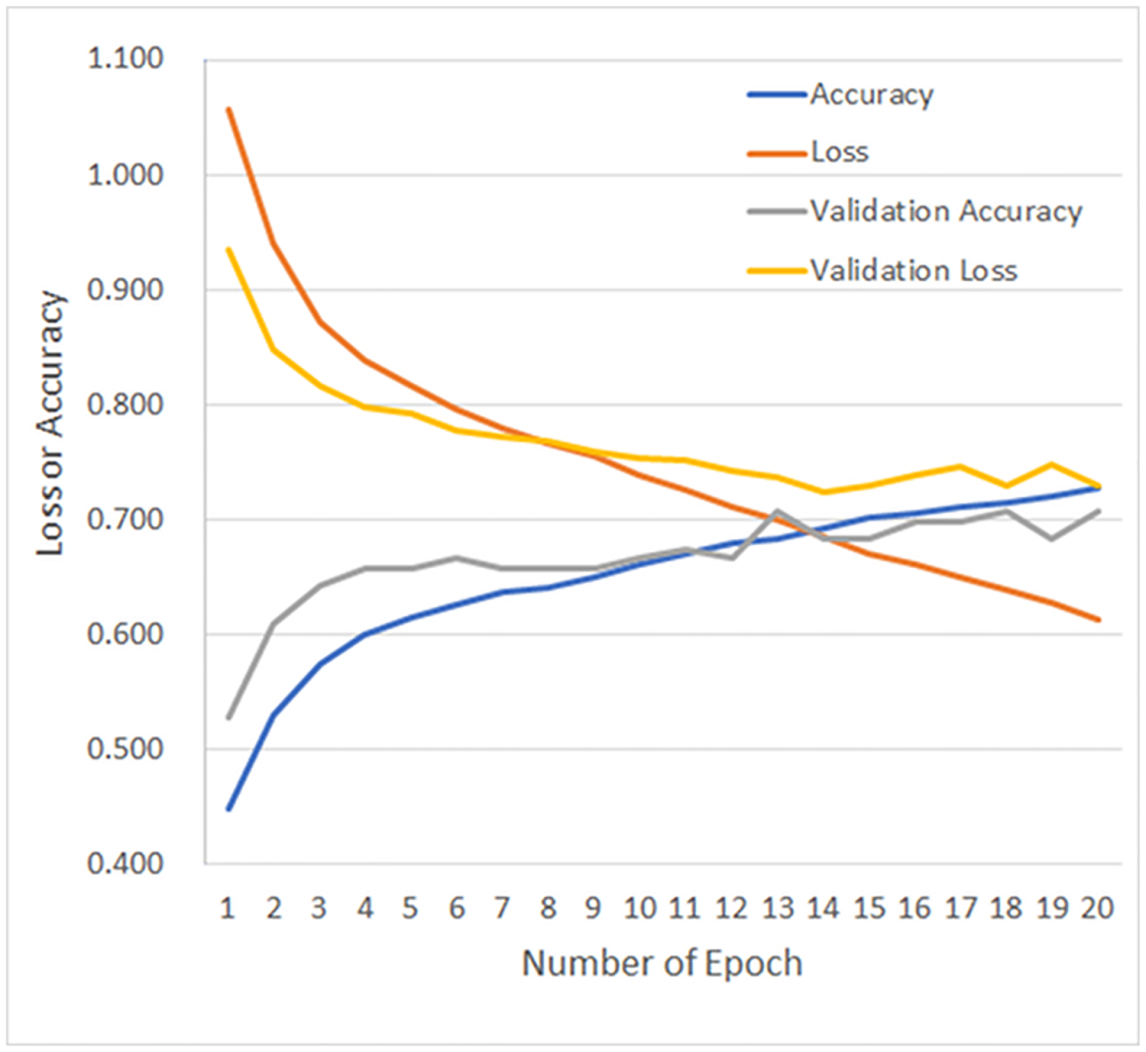
Representative results of loss and accuracy during DCNN training with transfer learning. Abbreviations: DCNN, deep convolutional neural network.

**Fig 5:**
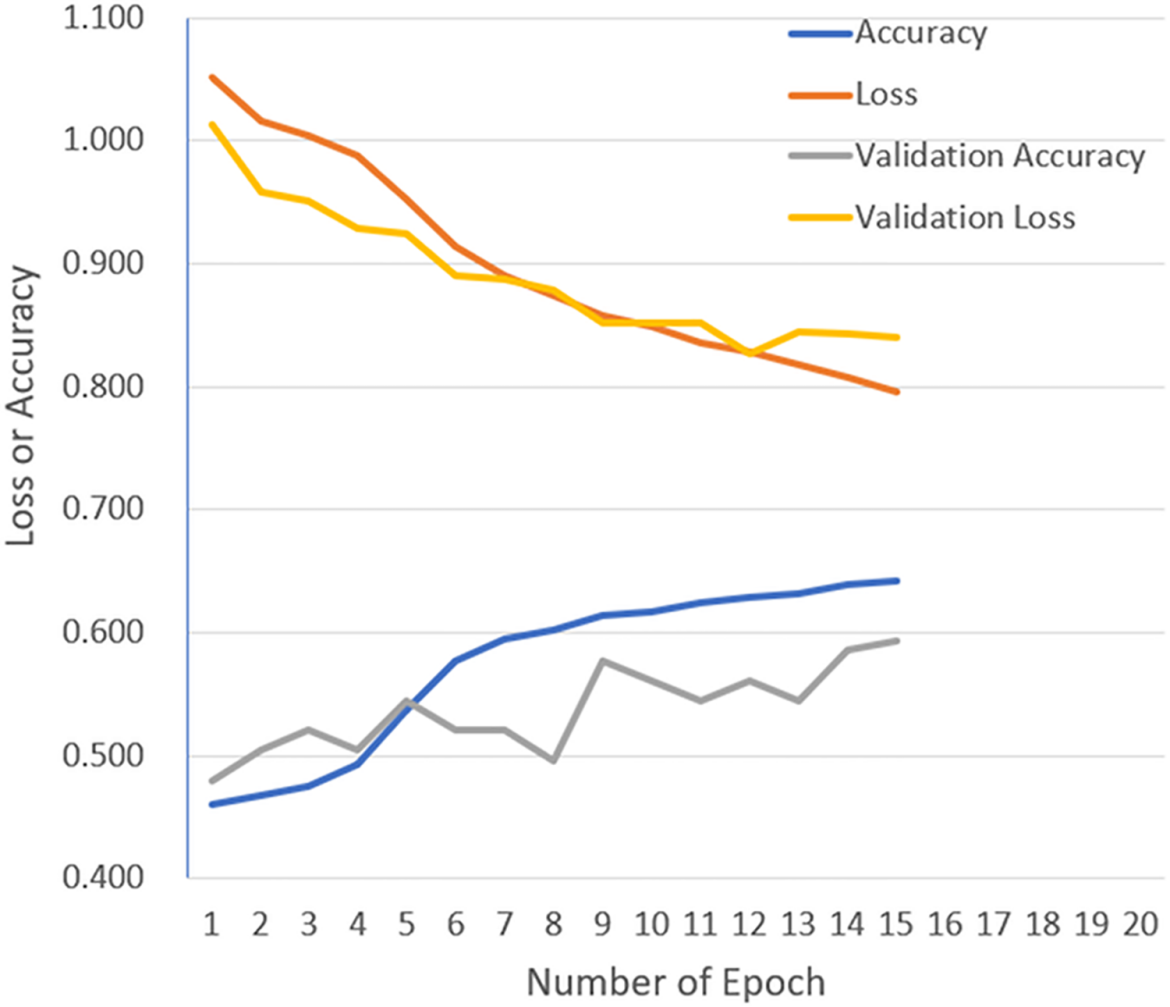
Representative results of loss and accuracy during DCNN training without transfer learning. Abbreviations: DCNN, deep convolutional neural network.

Figs 4 and 5 show representative results for loss and accuracy during DCNN training with and without transfer learning, respectively. Tables 3 and 4 show the corresponding confusion matrices between true labels and predicted labels obtained from CADx by DCNN with and without transfer learning, respectively. In addition, averaged confusion matrix was shown in Table 5, where the best averaged validation accuracy (68.0%) was obtained.

**Table 3:** Representative result of confusion matrix between true labels and predicted labels by DCNN with transfer learning.

		Predicted label		
		Benign nodule	Primary lung caner	Metastatic lung cancer
True label	Benign nodule	22	8	5
	Primary lung caner	6	46	8
	Metastatic lung cancer	5	4	19

Because splitting of the training and validation sets was random each time, the ratio between the 3 classes was variable. This confusion matrix corresponds to the results of Fig 4. Abbreviations: DCNN, deep convolutional neural network.

**Table 4:** Representative result of confusion matrix between true labels and predicted labels by DCNN without transfer learning.

		Predicted label		
		Benign nodule	Primary lung caner	Metastatic lung cancer
True label	Benign nodule	15	19	6
	Primary lung caner	10	43	6
	Metastatic lung cancer	6	3	15

Because splitting of the training and validation sets was random each time, the ratio between the 3 classes was variable. This confusion matrix corresponds to the results of Fig 5. Abbreviations: DCNN, deep convolutional neural network.

**Table 5:** Result of averaged confusion matrix between true labels and predicted labels by DCNN with transfer learning.

		Predicted label		
		Benign nodule	Primary lung caner	Metastatic lung cancer
True label	Benign nodule	19.9	12.6	7.2
	Primary lung caner	8.4	43.5	4.1
	Metastatic lung cancer	4.6	2.5	20.2

Because splitting of the training and validation sets was random each time, the ratio between the 3 classes was variable. This averaged confusion matrix was calculated from the 10 sets of classification results of 123 validation cases at the optimal hyperparameters. The validation accuracy of this confusion matrix was 68.0%. Abbreviations: DCNN, deep convolutional neural network.

## Discussion

The current results show that CADx of the ternary classification (benign nodule, primary lung cancer, and metastatic lung cancer) was better when using DCNN than when using the conventional method, and that transfer learning improved image recognition with the DCNN method. In addition, larger image sizes as inputs to DCNN improved the accuracy of lung nodule classification.

The averaged validation accuracies of CADx were 68.0% and 55.9% by the DCNN and conventional methods, respectively. These results confirm that DCNN was more useful for the CADx of lung nodules. While a major advantage of DCNN is that its performance for image recognition is superior to the conventional method, disadvantages are (i) that it is difficult to train because it frequently leads to overfitting and (ii) that large-scale data are needed for effective training. To prevent overfitting, we therefore used transfer learning to provide better diagnostic accuracy for lung nodules. We speculated that transfer learning was effective because our database was medium-scale (>1000 lung nodules).

The previous study [4] evaluated the performance of CADx without DCNN using the data for 1000 lung nodules obtained from our database. The study produced classification accuracies of 57.7% and 61.3% based on the conventional method and their proposed method (feature vectors calculated based on radiological findings), respectively. Because we used different methods for evaluating CADx performance, it was difficult to directly compare the performance with that of the previous study. However, according to both studies, the accuracy of CADx with the conventional method was nearly 60% for our database.

According to Litjens et al. [10], few studies have performed a thorough investigation of whether transfer learning gives better results for medical image analysis. Indeed, the results of two studies have left controversy about the efficacy of transfer learning [30,31]. By contrast, another two studies have shown that transfer learning with Google’s Inception v3 architecture can achieve diagnostic accuracy to expert human level in dermatology and ophthalmology [32,33]. In conjunction with the results of our study, CADx with transfer learning should improve diagnostic accuracy provided sufficient training data are used.

It was notable that image size (*L*) affected the accuracy of CADx by DCNN. Although image size is a simple factor, its effect on the accuracy of CADx was large in our study. Similar results were obtained in the previous study, where slice thickness of CT images could affect the detectability of CADe [34]. We speculated that, because VGG-16 was originally pretrained with an image size of 224 × 224, the best accuracy was obtained by finetuning VGG-16 with 2D CT images of the same size in our study. In the review of CAD by Litjens et al. [10], it was suggested that the exact architecture of deep learning was not the most important determinant of a good solution, and that data pre-processing or augmentation based on expert knowledge about the task could provide advantages beyond simply adding more layers to DCNN. Our results also show that a pre-processing step, such as adjusting the image size, should be performed carefully to obtain accurate results from CADx.

We developed a CADx method which classifies lung nodules into benign nodules, primary lung cancer, or metastatic lung cancer. A Lung CT Reporting and Data System (Lung-RADS) has been proposed for estimating lung cancer risk and the optimal follow-up strategy based on nodule-specific characteristics (i.e. nodule type, nodule size) [35]. Ciompi et al. developed CADx with DCNN for classifying the nodule type based on Lung-RADS [19]. However, although the nodule type is an important factor when evaluating lung cancer risk, it is not directly associated with pathological or clinical diagnosis. In contrast to this, our CADx method using DCNN can directly output the probabilities of the three classifications and would be more useful for clinicians than CADx which classifies nodule type.

Both our database and that of The Lung Image Database Consortium and Image Database Resource Initiative (LIDC/IDRI) [36] contain in excess of 1000 cases and CT images. However, clinical diagnostic results are only partially available in the LIDC/IDRI database. Few studies exist in which CADx was performed by DCNN with directly outputted probabilities of disease classification. We built our database to include both clinical diagnosis and radiological image findings [22].

There were several limitations to our study. First, we ignored all nodule-specific features, such as nodule size and type. The results of a previous study [4] show that CADx using radiological findings provided better results; given this, utilizing radiological findings may improve DCNN-based CADx. We hope that our study could serve as a basis for further exploration of CADx based on lung nodule characteristics. Second, we used 2D-DCNN for the CADx of lung nodules. Through image pre-processing, the 3D CT images of the lung nodules were converted to 2D CT images in three orthogonal planes, which greatly reduced the computational burden for DCNN training and testing. We focused on 2D-DCNN in the present study because it was difficult to perform transfer learning with 3D-DCNN on medical image analysis. We will attempt 3D-DCNN for CADx of lung nodules in a future study. Third, we only investigated the effect of smaller image sizes (*L* ≤ 224) because the computational cost precluded the evaluation of larger images. Given that the performance of graphic processing units has increased since the study inception, we expect to be able to evaluate the effect of larger image sizes in a future study.

In conclusion, the 2D-DCNN method was more useful for ternary classification of lung nodule than the conventional method for CADx, and transfer learning enhanced the image recognition for CADx by DCNN when using medium-scale training data. In addition, our results show that larger image sizes as inputs to DCNN improved the accuracy of lung nodule classification.

## Supporting information

S1 Table Raw results of CADx by DCNN with transfer learning in optimal hyperparameters. Raw results of CADx by DCNN with transfer learning in optimal hyperparameters.
(XLSX)

S2 Table Raw results of CADx by DCNN without transfer learning in optimal hyperparameters. Raw results of CADx by DCNN without transfer learning in optimal hyperparameters.
(XLSX)

S3 Table Averaged validation loss and validation accuracy of CADx by DCNN with transfer learning in all trials of random search. Averaged validation loss and validation accuracy of CADx by DCNN with transfer learning in all trials of random search.
(XLSX)

S4 Table Averaged validation loss and validation accuracy of CADx by DCNN without transfer learning in all trials of random search. Averaged validation loss and validation accuracy of CADx by DCNN without transfer learning in all trials of random search.
(XLSX)

S1 File Detail of conventional CADx and CADx by DCNN. Detail of conventional CADx and CADx by DCNN.
(DOCX)
