# Supplementary material for: Computer-aided diagnosis of lung nodule classification between benign nodule, primary lung cancer, and metastatic lung cancer at different image size using deep convolutional neural network with transfer learning
Source: PLoS One. 2018 Jul 27;13(7):e0200721. doi: 10.1371/journal.pone.0200721 (PMC6063408; doi:10.1371/journal.pone.0200721)
Supplement: S1 File — Detail of conventional CADx and CADx by DCNN. (DOCX) [file pone.0200721.s005.docx]

**S1 File**

**Detail of conventional CADx and CADx by DCNN**

**Software and hardware for DCNN**

We used Python-2.7 or Python-3.5 (https://www.python.org/), Keras (https://keras.io/) and Tensorflow (https://www.tensorflow.org/) with a Geforce GTX 980 and 1080 graphic processing unit to implement 2D-DCNN.

**Hyperparameters**

The following hyperparameters were used for conventional CADx [1, 2, 3, 4]:

- LBP-TOP had two hyperparameters, *LBP_R_* (with values of 1, 2, 3, 4, 5, 6, 7, and 8) and *LBP_P_* (with values of 8, 16, 24, 32, 40, 48, 56, and 64). *LBP_R_* is the distance between the center pixel and the neighbor pixel, and *LBP_P_* is the number of samples.
- For SVM*, C* (range, 2^−6^–2^12^) and **γ** (range, 2^−6^–2^12^) were used to control SVM with a radial basis function kernel.

We selected the best LBP-TOP and SVM hyperparameters by grid search [2].

The following hyperparameters were used for CADx with DCNN:

- *L* was the size of 2D CT images and was 56, 112, or 224.
- *B* was the number of batches, and was 50.
- *E* was the number of epochs when training DCNN and was 20, 25, or 30.
- *R* was the initial learning rate of stochastic gradient descent and was 0.00002 or 0.000025.
- *V* was the number of layers where parameters were not finetuned, and was 4, 7, or 11.
- *F* was the number of units in the FC layer, and was 384, 448, 512, 576, or 640.
- *D* was the strength of Dropout between the two FC layers and was 0.2, 0.4, or 0.6.

We performed random search to optimize these DCNN hyperparameters [5].

**Hyperparameter optimization in CADx by DCNN with transfer learning**

For the DCNN method, we performed a random search to optimize the hyperparameters, selecting the best DCNN hyperparameters. The number of random search trials was 25. To evaluate the effect of *L*, the following two steps were performed in random search. First, value of *L* was fixed to 56, 112, or 224. Then, the other hyperparameters were optimized using random search.

**Hyperparameter optimization in CADx by DCNN without transfer learning**

After selecting the best CADx hyperparameters for DCNN and transfer learning, training was repeated from the start, but without transfer learning. The values of *F* and *D* were fixed as the best obtained hyperparameters, and the values of *B* and *V* were set to 50 and 0, respectively. *E* and *R* were optimized by random search, but were selected from the following hyperparameters:

- The value of *E* was set as 10, 15, 20, 25, or 30.
- The value of *R* was set as 0.00005, 0.00007, 0.0001, 0.00015, or 0.0002.

The number of random search trials was 10 for DCNN training without transfer learning. *L* was fixed for the random search, as with transfer learning.

**References**

1. Chang C-C, Lin C-J. LIBSVM: a library for support vector machines. *ACM Trans Intell Syst Technol*. 2011;2(3):1-27. doi:10.1145/1961189.1961199.

2. Hsu C-W, Chang C-C, Lin C-J. A Practical Guide to Support Vector Classification. http://www.csie.ntu.edu.tw/. Accessed February 4, 2018.

3. Ojala T, Pietikainen M, Maenpaa T. Multiresolution gray-scale and rotation invariant texture classification with local binary patterns. *IEEE Trans Pattern Anal Mach Intell*. 2002;24(7):971-987. doi:10.1109/TPAMI.2002.1017623.

4. Ojala T, Pietikainen M, Harwood D. A comparative study of texture measures with classification based on featured distributions. *Pattern Recognit*. 1996;29(1):51-59. doi:10.1016/0031-3203(95)00067-4.

5. Bergstra J, Bengio Y. Random Search for Hyper-Parameter Optimization. *J Mach Learn Res*. 2012;13(Feb):281-305. http://www.jmlr.org/papers/v13/bergstra12a.html. Accessed June 29, 2017.
